# Detection and Independent Validation of Model-Based Quantitative Transcriptional Regulation Relationships Altered in Lung Cancers

**DOI:** 10.3389/fbioe.2020.00582

**Published:** 2020-06-10

**Authors:** Meiyu Duan, Haoqiu Song, Chaoyu Wang, Jiaxin Zheng, Hui Xie, Yupeng He, Lan Huang, Fengfeng Zhou

**Affiliations:** ^1^Key Laboratory of Symbolic Computation and Knowledge Engineering of Ministry of Education, College of Computer Science and Technology, Jilin University, Changchun, China; ^2^College of Computer Science, Hubei University of Technology, Wuhan, China; ^3^Key Laboratory of Symbolic Computation and Knowledge Engineering of Ministry of Education, College of Software, Jilin University, Changchun, China

**Keywords:** transcription factor, mRNA, quantitative measurement, regression, mqTrans

## Abstract

Differential expressions of genes are widely evaluated for the diagnosis and prognosis correlations with diseases. But limited studies investigate how transcriptional regulations are quantitatively altered in diseases. This study proposes a novel model-based quantitative measurement of transcriptional regulatory relationships between mRNA genes and Transcription Factor (TF) genes (mqTrans features). This study didn't consider the regulatory relationships between TF genes, so the mRNA genes were the protein-coding genes excluding the TF genes. The models are trained in the control samples in a lung cancer dataset and evaluated in two independent datasets and the hold-out testing samples from the third dataset. Twenty-nine mRNA genes are detected with transcriptional regulations quantitatively altered in lung cancers. The transcriptional modification technologies like RNA interference (RNAi) may be utilized to restore the altered transcriptional regulations in lung cancers.

## Introduction

Lung cancer is considered as the most prevalent cancer type and it is also one of the most frequent causes of cancer related death (Siegel et al., 2019, 2020). Even though, approved molecular targeted therapies other than chemotherapy are currently very limited, and the pathogenesis mechanism of lung cancer remains largely unclear (Amalraj et al., 2018). This disease may be impacted by both genetic mutations and environmental exposures (Ebben et al., 2016). Various precise diagnosis and prognosis models and technologies are under rapid developments (Jiang et al., 2019; Cheng et al., 2020; Wang et al., 2020).

Imaging technology has been widely used for the diagnosis and subtyping of lung cancers (Jacobsen et al., 2017; Zukotynski and Gerbaudo, 2017). Chest X-ray imaging is a popular way to screen for lung nodules, a major diagnosis factor of candidate lung cancer lesion (Huber et al., 2016). But its low resolution renders it difficult to find small nodules of the early-stage lung cancer and doesn't significantly improve the outcomes of lung cancers (Parkin and Pisani, 1996). Positron emission tomography (PET) is a functional imaging technology that can non-invasively observe the metabolic active locations throughout the animal body (Han et al., 2020). Magnetic resonance imaging (MRI) is another medical imaging technology for non-invasive cancer lesion detection without the radiological adverse effect of ionizing radiations (Momcilovic and Shackelford, 2018; Leandri et al., 2019). Computed tomography (CT) provides the high-resolution non-invasive imaging of the internal organs and serves as a very good technology to screen the early-stage cancers (Wang et al., 2019). Multi-modality fusion of different imaging technologies provides even more information for characterizing the cancer lesions, e.g. PET/CT (Zamboglou et al., 2019), etc.

Molecular biomarkers are usually evaluated for their individual associations with the disease risks and have been widely used in the decision processes of disease diagnosis and prognosis (He et al., 2019; Leiro et al., 2019; Yokobori et al., 2019; Muller et al., 2020). Transcription factor (TF) plays a critical role in cancer cell processes, such as cell proliferation, apoptosis, migration, and regulate gene expression. Thus, the identification and characterization of transcription factors involved in lung cancer will provide valuable information for further elucidation of the mechanism(s) underlying pathogenesis and the identification of potential therapeutic target types, which are critical for the development of therapeutic strategies. For example, the transcription factor gene E2F3 recognizes the DNA motifs of cell-cycle-associated genes by interacting with the retinoblastoma protein (pRB) and its differential expression in the human blood well-discriminates the lung cancer patients from the control samples (Al Ahmed and Nada, 2017).

Expression levels of various protein-coding genes demonstrate significant associations with the diagnosis or prognosis of lung cancers. The tyrosine kinase EGFR (Epidermal Growth Factor Receptor) binds to the epidermal growth factors on the cell surfaces and demonstrated differential expressions in the non-small cell lung cancers (NSCLC) (Rusch et al., 1993; Liang et al., 2012). EGFR's expression patterns are also associated with the survival of the NSCLC patients (Brabender et al., 2001). Another prognosis biomarker PD-L1 (Programmed Death-Ligand 1) demonstrates significant associations with the survival of multifocal lung cancers on the transcriptional level (Al Ahmed and Nada, 2017).

This study hypothesizes that the TF-mRNA regulatory modules altered in lung cancers may serve as diagnosis biomarkers and proposes a model-based quantitative metrics (mqTrans) to measure the TF-mRNA regulatory relationship using the machine learning algorithms. The proposed metrics mqTrans is applied to detect the TF-mRNA regulatory relationships in the healthy samples and this study focused on those mqTrans relationships that are significantly altered in the lung cancers. Most of the existing studies focused on investigating the differential expression or up-/down-regulation of a gene (Xin et al., 2020). But it's important to investigate the transcription regulation of healthy subjects (Busing et al., 2019; Rodriguez-Nunez et al., 2019). The metrics mqTrans is the first method to quantitatively measure the transcription regulation relationship. The observations are further validated by the hold-out testing samples from the above dataset and two independent datasets. Our experimental data suggests the existence of the quantitative TF-mRNA regulatory relationships statistically significantly altered in lung cancers, while the expression levels of TF or mRNA genes do not demonstrate significantly differential expressions.

## Materials and Methods

### Summary of the Training Dataset

The transcriptome dataset GSE19804 is chose to train the mqTrans models of TF-mRNA regulatory relationships (Lu et al., 2010, 2015). This transcriptome dataset recruits a cohort of 60 female Asian lung cancer patients with no smoking histories and 60 paired adjacent normal lung tissues as controls (dataset accession GSE19804), as shown in Table 1. This dataset is retrieved in the format Series Matrix File from the database Gene Expression Omnibus (GEO) (Clough and Barrett, 2016).

**Table 1:** Summary of the three datasets.

**Notation**	**Dataset**	**Samples**	**Features**	**Summary**
dsTrain	GSE19804	120	54,675	60 non-smoking female lung cancers vs. 60 lung normal
dsTest1	GSE30219	307	54,675	293 lung tumor samples vs. 14 non-tumoral lung samples
dsTest2	GSE33532	100	25,906	Four different sites (A, B, C, D) of individual primary tumors vs. matched distant normal lung tissue (N) from 20 patients

*These datasets are all methylomes generated by the GeneChip Human Genome U133 Plus 2.0 (platform GPL570) transcriptome array (Affymetrix, Inc.)*.

The total RNAs are isolated from the tissue specimens and the cDNA/cRNA are synthesized according to the standard Affymetrix protocol. Each sample is hybridized to the Gene Chip Human Genome U133 Plus 2.0 (platform GPL570) transcriptome array (Affymetrix, Inc). All probe sets of the previous version GeneChip Human Genome U133 Set are identically used on the GeneChip Human Genome U133 Plus 2.0 Array. The sequences of these probe sets were derived from the databases GenBank™, dbEST, and RefSeq. The sequence clusters were created from the UniGene database (Build 133, April 20, 2001) and then refined by analysis and comparison with a number of other publicly available databases, including the Washington University EST trace repository and the University of California, Santa Cruz Golden-Path human genome database (April 2001 release).

Each sample generated by the platform GPL570 has 54,675 features with the normalized probeset values. The 54,675 transcriptomic features of the platform GPL570 are annotated with human gene symbols and split into two groups, i.e., TF and mRNA. This study didn't consider the regulatory relationships between TF genes, so the mRNA genes investigated in this study are the protein-coding genes excluding the TF genes. We eliminate the features without the gene symbol annotation. Among the remaining 45,056 features, there are 5,827 TF features, whose gene symbols are annotated as transcription factors based on the database AnimalTFDB version 3.0 (Hu et al., 2019). The other 39,229 transcriptomic features are regarded as the mRNA features, which are screened for their quantitative regulations by the TF features.

### Two Independent Validation Datasets

Two more transcriptome datasets generated using the same microarray platform GPL570 are chosen to independently validate the model-based quantitative metrics of transcriptional regulatory relationships (mqTrans) features, as shown in Table 1. The dataset GSE30219 is designed to detect the metastasis-prone tumors and has 293 lung tumor samples and 14 non-tumoral lung samples (Rousseaux et al., 2013). This dataset provides the sample set of transcriptomic features. The other validation dataset GSE33532 collects four tumor lesion sites and one matches normal lung tissue from each of the 20 lung cancer patients (Meister et al., 2014). The authors of this dataset carry out a stringent pre-screening step to remove those probesets with minor expression levels or no matching expressed transcripts, etc. The expression matrix provided by this study has 25,906 features.

All the three transcriptomic datasets in Table 1 are generated on the same microarray platform GPL570, and their transcriptomic features have the same annotations.

### Formulation of mRNA Features Regulated by Multiple Transcription Factors

This study hypothesizes that one mRNA gene may be transcriptionally regulated by multiple transcription factors. In a transcriptomic dataset, a feature is regarded as either a transcription factor (TF) or an mRNA gene (mRNA). So the expression level of an mRNA feature is modeled as$mRNA^{\prime }\left(i\right)=w_{0}+\overset{5827}{\underset{k=1}{\sum }}w\left(k,i\right)\times TF\left(k\right)$, where *i* is an integer between [1, 39,229] and the weight of the *k*^*th*^ transcription factor TF(*k*) is *w*(*k, i*).

This study calculates the TF weights using the L1-regularized regression as implemented by the function “Lasso()” in the Python package sklearn.linear_model. Because an mRNA gene is not regulated by all the 5,827 TF genes, the model consists of both feature selection and regression. Lasso is one of the most efficient ways to provide both functionalities. The TF features are regarded as being selected if their weights are non-zero. The default parameter values are used. Lasso() generated ZERO weights for many features and only those features with non-zero weights are kept for further analysis. This study assumes that only the TF genes with non-zero weights regulate the target mRNA genes.

### Model-Based Quantitative Metrics of TF-mRNA Regulatory Relationships (mqTrans)

This study defines the absolute difference between *mRNA*′(*i*) and *mRNA*(*i*) as the mqTrans feature of the original *ith* mRNA feature, where *mRNA*′(i) is defined as above and *mRNA*(*i*) is the real expression level of the *i*^*th*^ mRNA feature. For the simplicity of notations, the calculated and real expression values of a given transcriptomic feature F are also denoted as *mRNA*′(*F*) and *mRNA*(*F*), respectively. The model-based quantitative metrics of the TF-mRNA regulatory relationship of the mRNA feature F is defined as *mqTrans*(*F*) = |*mRNA*(*F*) − *mRNA*′(*F*)|.

### Experimental Design

It's difficult to find multiple datasets on the same disease type and the same technological platform. All the three datasets were the case-control studies with reasonable numbers of samples. The model training relies on the high-quality training data. The dataset dsTrain (GSE19804) consists of the most balanced sample classes compared with the other two datasets, as shown in Table 1. But the dataset dsTrain collected the female samples from Taiwan only, which may cause the model bias. So the regression models trained over the dataset dsTrain need to be evaluated by the hold-out samples in the same dataset and the independent validation datasets, i.e., dsTest1 and dsTest2 in this study. Due to the nature of biomedical research, it's difficult to collect the healthy lung tissues. So the two datasets dsTest1 and dsTest2 have imbalanced numbers of disease and control samples. The statements in this study may be further validated with future balanced datasets.

**Figure 1:**
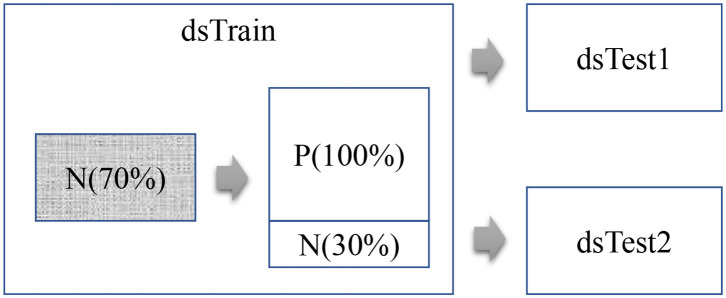
Flowchart of this study. This study uses three datasets, i.e., Train, Test1, and Test2. The regression model of each mRNA feature is trained over the 70% randomly-chosen N samples in the dataset Train.

This study firstly trains a Lasso regression model for each mRNA feature *F* using the randomly-chosen 70% of the healthy control samples from the dataset dsTrain, as shown in Figure 1. The lung cancer samples and the controls are denoted as P and N samples, respectively. Then the model is evaluated on the remaining 30% of the N samples and all the P samples of the dataset dsTrain, as denoted in Table 1. The two independent validation datasets dsTest1 and dsTest2 are utilized to further evaluate the TF-mRNA regulatory relationships detected in the N samples of the dataset Train.

So the engineered feature *mqTrans*(*F*) = |*mRNA*(*F*) − *mRNA*′(*F*)| tends to be close to zero, if the validation samples quantitatively maintain a similar TF-mRNA regulatory relationship as in the model-training samples.

## Results and Discussion

### Summary of the mqTrans Features in the Dataset Train

This study constructs the regression models for 2,463 out of the 39,299 mRNA features. Each regression model has at least one TF feature with a non-zero weights. And the non-zero weights for the TF features suggest that these TF features have associations with the expression levels of the specific mRNA feature.

Two popular criteria are utilized to ensure the regression qualities. The Pearson correlation coefficient (PCC) is calculated between the original values “mRNA()” and the regression-based calculates values “mRNA′()” of each mRNA feature in the training dataset. The Pvalue of the false discovery rate (FDR) is also calculated by the Python package Lasso() to ensure the statistical significance of FDR estimation. Only those features with PCC >0.5 and *P* < 0.05 are kept for further analysis (Amalraj et al., 2018). Other criteria may also be utilized to evaluate the regression models from the other aspects of views, e.g., the root mean square difference (RMSD) (Peslin et al., 1992).

The regression models of 2,030 mRNA features pass the above screening criteria. For the convenience of discussion, the mqTrans feature of the original mRNA feature *F* is also annotated using the original feature name *F*.

### The mqTrans Features Altered in Lung Cancers

The mqTrans feature F in the cancer samples is supposed to be larger than that in the controls on average. The mqTrans feature F describes the absolute difference between the real expression level and the regression model-based predicts expression level of the feature F. If a group of samples share a similar transcriptional regulatory relationship of the feature F with the randomly-chosen 70% of the control samples in the dataset dsTrain, the calculated mqTrans feature F would be close to zero. And if the transcriptional regulatory relationship of the mRNA F is altered in the lung cancer samples, the mqTrans feature F in the lung cancers would be larger than that in the control samples.

**Figure 2:**
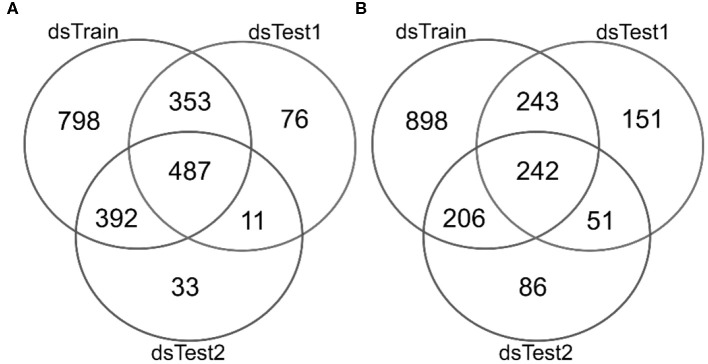
Venn plots of the detected mqTrans features in three datasets dsTrain, dsTest1 and dsTest2. The numbers of detected mqTrans features in the three datasets **(A)** before and **(B)** after the screening criteria of mqTrans features alter in lung cancers. The Venn plot is generated using the online software from Prof. Yves Van de Peer's lab, which is available at http://bioinformatics.psb.ugent.be/webtools/Venn/.

There are 2,030 mqTrans features detected in the dataset dsTrain, and 487 of them are confirmed in the two independent validation datasets dsTest1 and dsTest2, as shown in Figure 2A. 1,589 mqTrans features in the dataset dsTrain demonstrate alternations in the lung cancer samples, as screened in the above. The other two independent validation datasets have 687 and 585 mqTrans features altered in lung cancers, respectively. The overlap of these three feature lists have 242 mqTrans features, as shown in Figure 2B.

### Restrict the FDRs of the mqTrans Features

**Figure 3:**
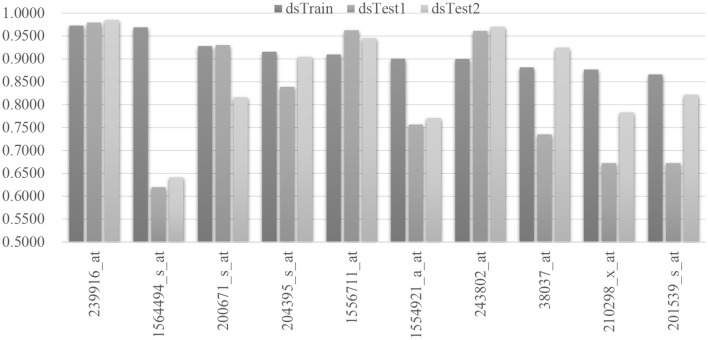
Pearson correlation coefficients of the regression performances of the 10 mqTrans features. We select the top 10 mqTrans features with the largest Pearson correlation coefficients on the hold-out testing dataset. The regression model of each mqTrans feature is evaluated for its regression performances in the control samples in the three datasets dsTrain, dsTest1 and dsTest2.

The false discovery rate (FDR) is calculated for the regressed model of each mRNA feature, and the mqTrans features with FDR ≥ 0.01 are excluded from further analysis. There are 76 mqTrans features passed the screening, and 29 of them are confirmed in the other two independent validation datasets dsTest1 and dsTest2. Figure 3 shows the top 10 mqTrans features ranked by the largest Pearson correlation coefficients (PCC) and illustrates the PCC values of these mqTrans features are consistently large in all the three datasets. The Pearson correlation coefficients of all the 29 mqTrans in three datasets are shown in the Supplementary Figure 1. The mqTrans feature 239916_at is within the gene CFAP52 (Cillia and flagella associated protein 52) and achieves 0.9729, 0.9791 and 0.9851 in the three datasets dsTrain, dsTest1 and dsTest2, respectively.

**Figure 4:**
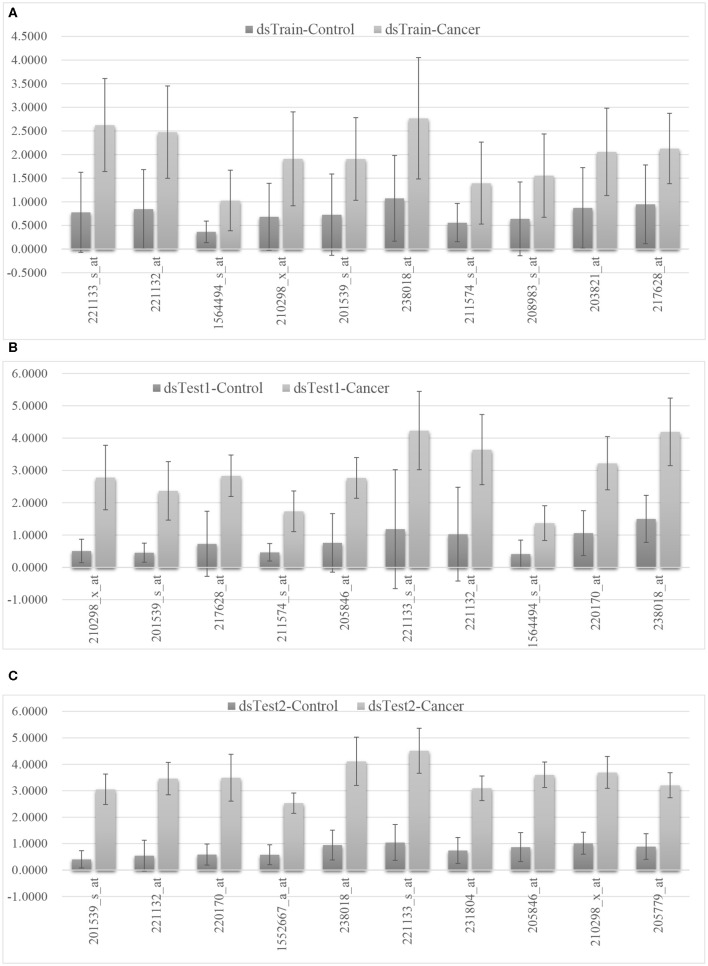
The distributions of the 10 mqTrans features in the control and lung cancer samples. The mean and standard deviation values of the calculated mqTrans features in the dataset **(A)** dsTrain, **(B)** dsTest1, and **(C)** dsTest2. The top 10 mqTrans features were ranked by the ratio of the mean value of mqTrans features between the cancer group and the control group.

We selected the mqTrans features with the top 10 largest ratios of the mean values of mqTrans features in the cancer group and the control group in three data sets and the detail is shown in the Figure 4. The 29 mqTrans features are calculated based on the regression models trained on the randomly-chosen 70% of the control samples in the dataset dsTrain, and satisfyingly demonstrate consistently small values in the other control samples in the same dataset and the two independent validation datasets, as shown in the Supplementary Figure 2. The engineered mqTrans features are at much higher levels in the lung cancer samples in all the three datasets. So the experimental data suggest that these 29 mRNA features have quantitatively altered transcriptional regulatory relationships in the lung cancer samples. For example, the above-discussed mqTrans feature 201539_s_at has much larger values in the lung cancer samples than in the control ones, as shown in Figure 4.

### Literature Support for the Detected Transcriptional Regulatory Relationships Altered in Lung Cancers

**Figure 5:**
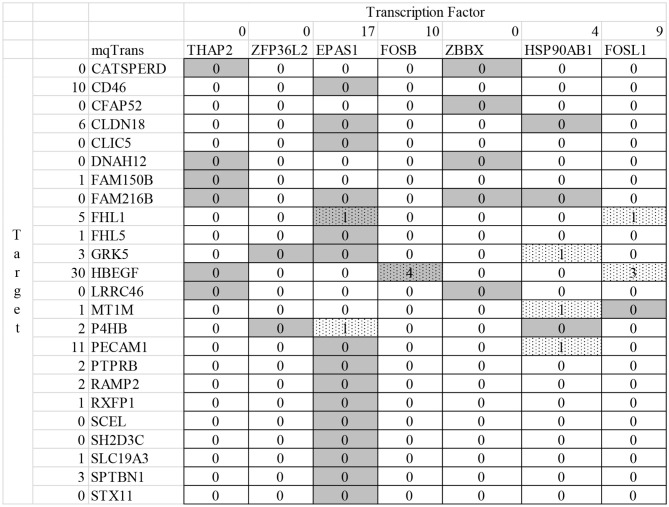
Summary of the literature support for the detected transcriptional regulatory relationships altered in lung cancers. The heatmap with the solid boundary lines illustrate the transcriptional regulatory relationships altered in lung cancers. Each row is a target mRNA, which is transcriptionally regulated by the transcription factors denoted by the gray boxes in that row. The dotted box suggests that the transcription factor in that column and the target mRNA in that row are investigated in the same PubMed literature (Canese and Weis, 2013), and the number in that box is the number of publications mentioned both the transcription factor and the target mRNA. The number in the column left to the target mRNA gene symbols is the number of publications investigating the gene symbol of the target mRNA in lung cancers. And the number in the second row is the number of publications investigating the TF gene under the number.

The numbers in the heatmap region of Figure 5 suggest that limited studies have investigated the transcriptional regulatory relationships described by the detected mqTrans features. The screening criteria is to find a publication in the database PubMed mentioning both the target mRNA and the TF in the title or abstract (Canese and Weis, 2013). And the screening is carried out on February 3, 2020. The expression level of the target mRNA gene CFAP52 is accurately predicted in the dataset dsTrain and further independently supported by two other datasets dsTest1 and dsTest2, as shown in Figure 2. The ratios between the averaged mqTrans features in the lung cancer and control samples are 1.6215, 1.8015, and 1.8522 in the three datasets dsTrain, dsTest1, and dsTest2, respectively. This suggests that the transcriptional regulatory relationship between CFAP52 and the TF ZBBX is quantitatively changed in lung cancers compared with the control samples.

Neither CFAP52 nor ZBBX is investigated for the relationship with lung cancer, as illustrated y 0 in the second row and the second column in Figure 5. A gene is defined to be investigated by a publication if its title and abstract has this gene symbol and the disease name, i.e., “lung cancer” or “lung tumor” or “lung adenocarcinoma” or “lung squamous cell carcinoma.” The literature research is carried out on February 3, 2020. The TF ZBBX and the target mRNA CFAP52 are mentioned together as downregulated genes in nasopharyngeal carcinoma (Ye et al., 2019).

The target mRNA HBEGF (heparin binding EGF like growth factor) is transcriptionally regulated by the TF FOSB (FosB Proto-Oncogene, AP-1 Transcription Factor Subunit) and their transcriptional regulatory relationship is quantitatively changed in lung cancers. The regression model of HBEGF is trained in the randomly-chosen 70% control samples from the dataset dsTrain and the model is supported by the other samples in the datasets dsTrain, dsTest1, and dsTest2, with Pearson correlation coefficients ranging between 0.6936 and 0.9246. The mqTrans features are 2.1529, 2.2641, and 2.7139 times in lung cancers compared with the control samples in the three datasets. HBEGF and FOSB are separately investigated for their connections with lung cancers, and they are investigated together only in one study for their upregulated in the hypospadias patients (Karabulut et al., 2013).

### Biological Inferences of the Detected Transcriptional Regulatory Relationships

This study firstly uses the online enrichment analysis platform DAVID (Huang da et al., 2009a,b) to screen for the enriched functions in the 24 target mRNA genes and 7 regulatory TF genes as shown in Figure 5 and Table 2. Unfortunately, no enriched functions are detected in the target mRNA genes or the TF genes, respectively.

**Table 2:** Annotations of the detected mqTrans biomarkers with quantitatively altered transcriptional regulations in lung cancers.

**mqTrans**	**Gene**	**Location**	**CelLoc**	**Annotation**
205846_at	PTPRB	chr12q15-q21	Membrane	Protein tyrosine phosphatase, receptor type, B
210190_at	STX11	chr6q24.2	Membrane	Syntaxin 11
217546_at	MT1M	chr16q13	Nucleus, cytoplasm	Metallothionein 1M
210298_x_at	FHL1	chrXq26	Nucleus, cytoplasm	Four and a half LIM domains 1
200671_s_at	SPTBN1	chr2p21	Membrane; cytoskeleton	Spectrin, beta, non-erythrocytic 1
220170_at	FHL5	chr6q16.1-q16.3	Nucleus	Four and a half LIM domains 5
205779_at	RAMP2	chr17q12-q21.1	Membrane	Receptor (G protein-coupled) activity modifying protein 2
203821_at	HBEGF	chr5q23	Membrane; extracellular space	Heparin-binding EGF-like growth factor
1556711_at	FAM216B	chr13q14.11	-	Family with sequence similarity 216, member B
1552715_a_at	RXFP1	chr4q32.1	Membrane	Relaxin/insulin-like family peptide receptor 1
230601_s_at	LRRC46	chr17q21.32	-	Leucine rich repeat containing 46
208983_s_at	PECAM1	chr17q23.3	Membrane; Cell junction	Platelet/endothelial cell adhesion molecule 1
221132_at	CLDN18	chr3q22.3	Membrane; tight junction	Claudin 18
201539_s_at	FHL1	chrXq26	Nucleus; Cytoplasm	Four and a half LIM domains 1
238018_at	FAM150B	chr2p25.3	Secreted	Family with sequence similarity 150, member B
217628_at	CLIC5	chr6p12.3	Membrane; cytoskeleton; cytoplasm; cell cortex	Chloride intracellular channel 5
38037_at	HBEGF	chr5q23	Membrane; extracellular space	Heparin-binding EGF-like growth factor
1554921_a_at	SCEL	chr13q22	Membrane; Cytoplasm	Sciellin
237020_at	CATSPERD	chr19p13.3	Membrane	Catsper channel auxiliary subunit delta
220736_at	SLC19A3	chr2q37	Membrane	Solute carrier family 19 (thiamine transporter), member 3
1555191_a_at	FHL5	chr6q16.1-q16.3	Nucleus	Four and a half LIM domains 5
221133_s_at	CLDN18	chr3q22.3	Membrane; tight junction	Claudin 18
243802_at	DNAH12	chr3p14.3	Cytoskeleton	Dynein, axonemal, heavy chain 12
231804_at	RXFP1	chr4q32.1	Membrane	Relaxin/insulin-like family peptide receptor 1
1564494_s_at	P4HB	chr17q25	Membrane; Endoplasmic reticulum; Melanosome	Prolyl 4-hydroxylase, beta polypeptide
211574_s_at	CD46	chr1q32	Membrane	CD46 molecule, complement regulatory protein
204395_s_at	GRK5	chr10q26.11	Membrane; Nucleus; Cytoplasm	G protein-coupled receptor kinase 5
239916_at	CFAP52	chr17p13.1	Cytoplasm; flagellum	Cilia and flagella associated protein 52
1552667_a_at	SH2D3C	chr9q34.11	Membrane; Cytoplasm	SH2 domain containing 3C

*Column “mqTrans” is the target mRNA feature name from the transcriptome microarray platform GPL570. Columns “Gene” and “Location” are the corresponding gene symbol and the chromosome locations in the Human Reference Genome GRCh37 released in February 2009. Column “CelLoc” is the sub-cellular localizations of the proteins encoded by this gene, and the annotation is collected from the database UniProt Knowledgebase. Column “Annotation” is the functional annotation collected from the definition file of the microarray platform GPL570*.

But the sub-cellular localization annotations from the database UniProt Knowledgebase (UniProt, 2019) demonstrate an enrichment of membrane-associated proteins encoded by these biomarker genes, as shown in Table 2. Nineteen out of the 24 target mRNA genes encoded membrane-associated proteins. Nucleus (6) and cytoplasm (8) are another two common sub-cellular locations for the proteins encoded by the genes with transcriptional regulations altered in lung cancers.

EPAS1 is known to be a major transcriptional regulator associated with the diagnosis and prognosis of lung cancers (Wang et al., 2018; De Bastiani and Klamt, 2019; Zhang et al., 2019). This study detects that sixteen EPAS1-regulated genes demonstrated quantitatively altered transcriptional regulations in lung cancers. But none of these 16 genes are investigated together with EPAS1 in lung cancers, by screening for their co-appearances in the PubMed literature (Canese and Weis, 2013). The experimental data in this study suggest that these 16 genes had similar quantitative correlations with the transcription factor EPAS1 on the expression level in the control samples in the two independent datasets and the hold-out testing dataset, while such correlations are altered in the lung cancer samples in these three datasets.

### Cross-Dataset Confirmation of the mqTrans Features in Lung Cancer

We further use the control samples in the other two datasets to train the mqTrans features and validate the 29 mqTrans biomarkers detected in the above sections. The dataset GSE19804 has only the female samples from Taiwan, which may cause the sex and ethnicity bias. Both of the other two datasets GSE30219 and GSE33532 consist of both male and female samples. The dataset GSE30219 was collected from the French cohort, while the dataset GSE33532 was from the Germany cohort. So the control samples in these two datasets were used to train the mqTrans models, which were validated for their alternations in lung cancers.

Three out of the 29 lung cancer mqTrans biomarkers are further confirmed by all the four validation experiments, as shown in Table 3. The mqTrans models trained using the control samples of the dataset GSE30219 confirmed 7 and 8 out of the 29 mqTrans biomarkers detected in the above sections. And there are six mqTrans biomarkers confirmed by both of these two experiments. There are 6 and 9 GSE33532-trained mqTrans models confirmed by the datasets GSE19804 and GSE30219, respectively. Both of the two experiments support five mqTrans biomarkers.

**Table 3:** The 29 lung cancer mqTrans biomarkers confirmed by the cross-dataset validations.

**Training**	**GSE30219**		**GSE33532**	
**Testing**	**GSE19804**	**GSE33532**	**GSE19804**	**GSE30219**
**Lung cancer specific mqTrans features**	237020_at	**238018_at**	**238018_at**	**238018_at**
	**243802_at**	203821_at	1556711_at	237020_at
	**239916_at**	38037_at	237020_at	**243802_at**
	203821_at	217546_at	**243802_at**	217546_at
	217546_at	1555191_a_at	**239916_at**	38037_at
	1556711_at	**243802_at**	230601_s_at	**239916_at**
	**238018_at**	1556711_at		230601_s_at
		**239916_at**		1554921_a_at
				203821_at

*The mqTrans models are trained using the control samples in the dataset identified in the first row. The validation dataset is given in the second row. The mqTrans biomarkers confirmed in all the four validation experiments are highlighted in bold*.

The three mqTrans biomarkers confirmed by all the six validation experiments are 238018_at (FAM150B), 243802_at (DNAH12) and 239916_at (CFAP52). Each validation experiment trains the mqTrans models using one dataset and validates the models using another one. The two genes DNAH12 and do not have literature supports for their associations with lung cancer, but FAM150B was observed to be potent ligands for human anaplastic lymphoma kinase (ALK) (Guan et al., 2015), whose aberrant activation is involved in non-small cell lung cancers (Hallberg and Palmer, 2013).

There are only three mqTrans biomarkers supported by these four cross-dataset validation experiments. This is mainly due to that the numbers of the control samples are only 14 (out of 307 total samples) and 20 (out of 100 total samples) in the two datasets GSE30219 and GSE33532.

## Conclusion

This study proposes a novel model-based quantitative measurement mqTrans of the transcriptional regulatory relationship between mRNA and TF, and utilizes the mqTrans features to detect 29 transcriptional regulatory relationships altered in lung cancers. The conclusions are validated using both the samples in the same dataset and two independent datasets. These 29 mqTrans biomarkers of lung cancers may be verified for their diagnosis and prognosis roles by various biological knock-down technologies. Three out of the 29 mqTrans biomarkers are further confirmed by the cross-dataset validation experiments. One of the three mqTrans biomarker genes encodes a ligand for human kinase ALK, which is involved in the non-small cell lung cancers.

It's difficult to collect healthy lung tissues as the control samples. So the two independent validation datasets do not have balanced numbers of control samples. The statements in this study may be further validated in the future balanced datasets.

## Data Availability Statement

The datasets generated during the current study are available in the GEO database, https://www.ncbi.nlm.nih.gov/geo/. The gene annotation file is download from https://www.ncbi.nlm.nih.gov/geo/query/acc.cgi?acc=GPL570. The gene symbols are annotated as transcription factors based on the database AnimalTFDB version 3.0 (http://211.67.31.242/HumanTFDB/).

## Author Contributions

FZ: conceptualization, supervision, and project administration. FZ and MD: methodology. MD, HS, HX, and YH: software. FZ, MD, HS, CW, JZ, HX, YH, and LH: formal analysis. MD: data curation. FZ, MD, CW, and JZ: investigation. FZ, MD, HS, HX, and YH: writing—original draft. FZ, MD, HS, and LH: writing—review and editing. MD and LH: visualization. FZ and LH: funding acquisition.

## Conflict of Interest

The authors declare that the research was conducted in the absence of any commercial or financial relationships that could be construed as a potential conflict of interest.
