# Supplementary material for: Detection and Independent Validation of Model-Based Quantitative Transcriptional Regulation Relationships Altered in Lung Cancers
Source: Front Bioeng Biotechnol. 2020 Jun 10;8:582. doi: 10.3389/fbioe.2020.00582 (PMC7325891; doi:10.3389/fbioe.2020.00582)
Supplement: Supplementary file 1 [file Image_1.pdf]

Detection and independent validation of model-based quantitative transcriptional regulation relationships altered in lung cancers

Meiyu Duan[1], Haoqiu Song[2,1], Chaoyu Wang[3], Jiaxin Zheng[3], Hui Xie[1], Yupeng He[3], Lan Huang[1] and Fengfeng Zhou[1,*]
1 College of Computer Science and Technology, and Key Laboratory of Symbolic Computation and Knowledge Engineering of Ministry of Education, Jilin University, Changchun, Jilin 130012, China;
2 College of Computer Science, Hubei University of Technology, Wuhan, Hubei 430068, China;
3 College of Software, and Key Laboratory of Symbolic Computation and Knowledge Engineering of Ministry of Education, Jilin University, Changchun, Jilin 130012, China;

# Correspondence: author: Fengfeng Zhou, e-mail: FengfengZhou@gmail.com or ffzhou@jlu.edu.cn . Web site: http://www.healthinformaticslab.org/.

Emails:
Meiyu Duan: 2257770119@qq.com
Haoqiu Song: song4987@163.com
Chaoyu Wang: 2651093812@qq.com
Jiaxin Zheng: 604799676@qq.com
Hui Xie: xiehui2116@mails.jlu.edu.cn
Yupeng He: heyp5516@mails.jlu.edu.cn
Lan Huang: huanglan@jlu.edu.cn
Fengfeng Zhou: FengfengZhou@gmail.com

Supplementary Figure 1

Pearson correlation coefficients of the regression performances of the 29 mqTrans features. The regression model of each mqTrans feature is evaluated for its regression performances in the control samples in the three datasets dsTrain, dsTest1 and dsTest2.

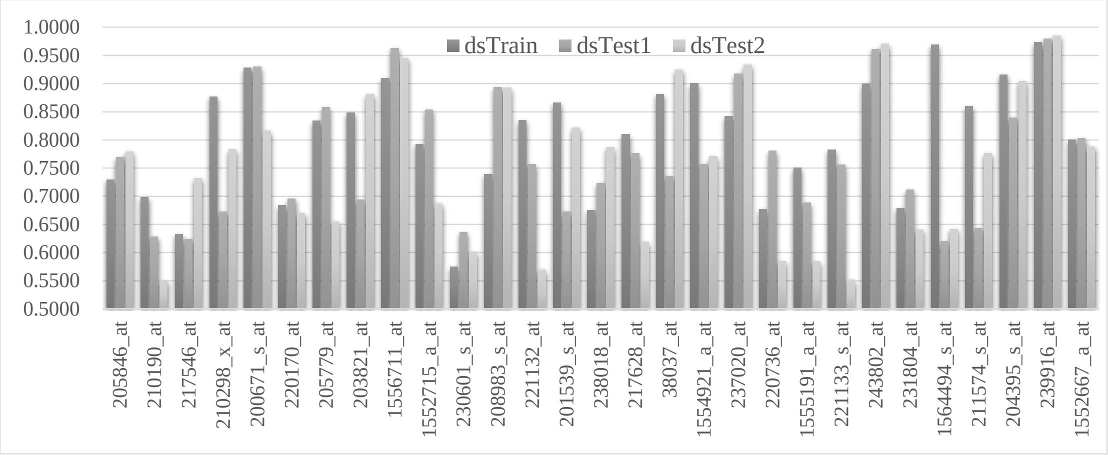

Supplementary Figure 2

The distributions of the 29 mqTrans features in the control and lung cancer samples. The mean and standard deviation values of the calculated mqTrans features in the dataset (a) dsTrain, (b) dsTest1 and (c) dsTest2.

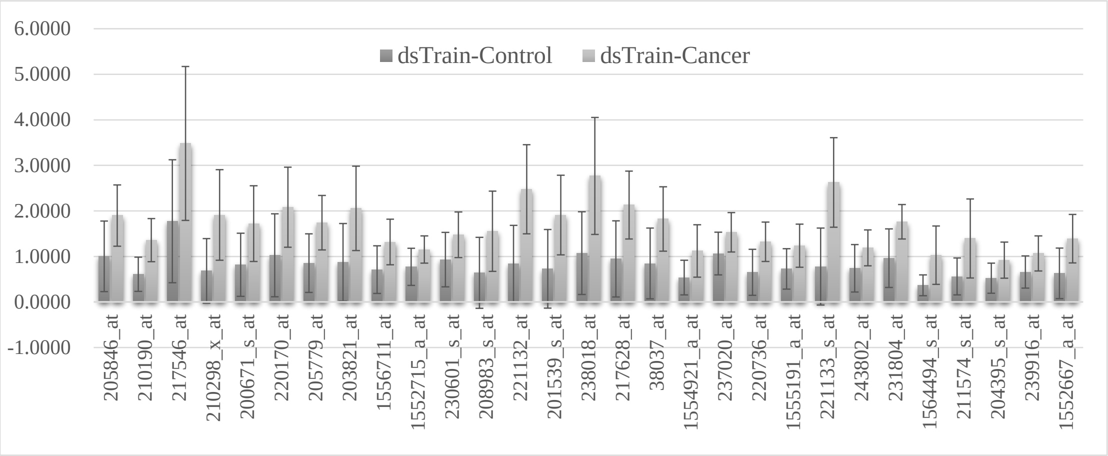

(a)

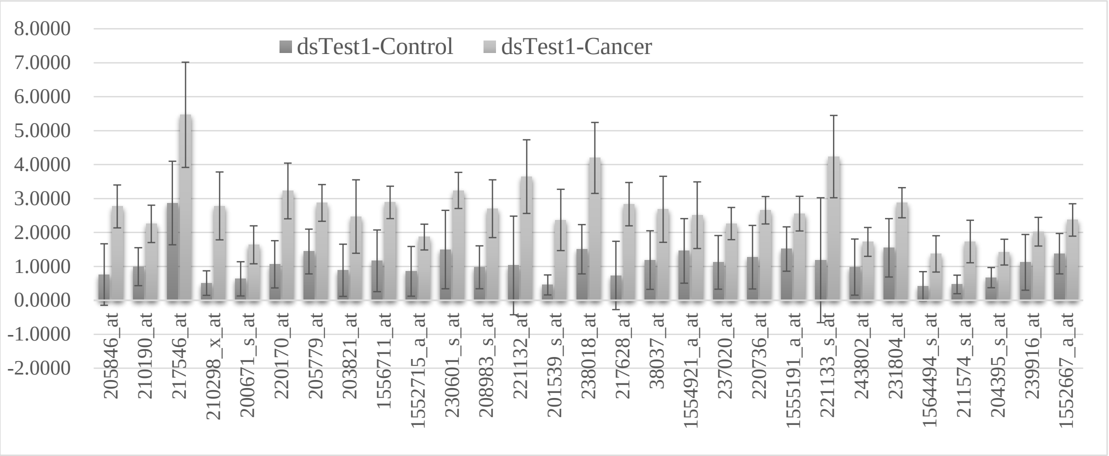
8.0000
7.0000
6.0000
5.0000
4.0000
3.0000
2.0000
1.0000
0.0000
-1.0000
-2.0000
dsTest1-Control
dsTest1-Cancer
205846_at
210190_at
217546_at
210298_x_at
200671_s_at
220170_at
205779_at
203821_at
1556711_at
1552715_a_at
230601_s_at
208983_s_at
221132_at
201539_s_at
238018_at
217628_at
38037_at
1554921_a_at
237020_at
220736_at
1555191_a_at
221133_s_at
243802_at
231804_at
1564494_s_at
211574_s_at
204395_s_at
239916_at
1552667_a_at

(b)

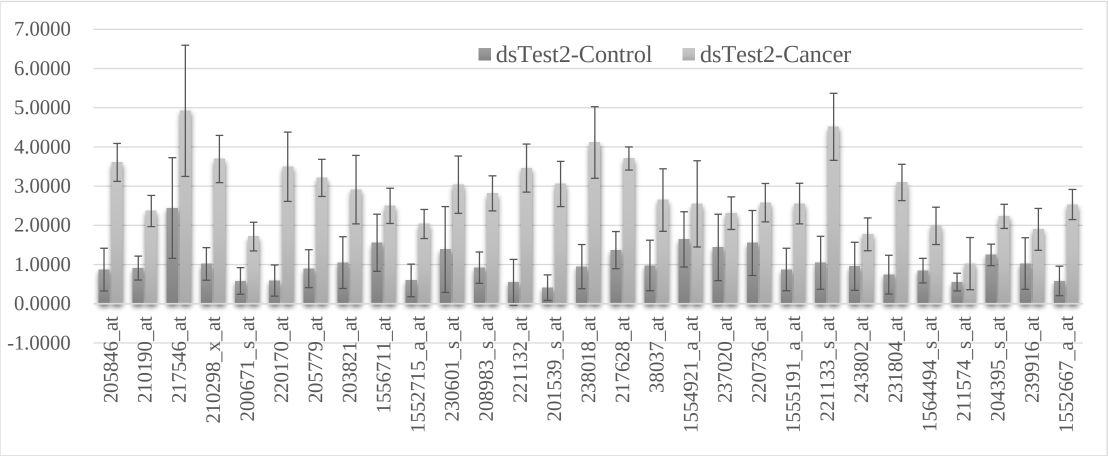
7.0000
6.0000
5.0000
4.0000
3.0000
2.0000
1.0000
0.0000
-1.0000
dsTest2-Control
dsTest2-Cancer
205846_at
210190_at
217546_at
210298_x_at
200671_s_at
220170_at
205779_at
203821_at
1556711_at
1552715_a_at
230601_s_at
208983_s_at
221132_at
201539_s_at
238018_at
217628_at
38037_at
1554921_a_at
237020_at
220736_at
1555191_a_at
221133_s_at
243802_at
231804_at
1564494_s_at
211574_s_at
204395_s_at
239916_at
1552667_a_at

(c)
